# Free Vibrations of a Cantilevered SWCNT with Distributed Mass in the Presence of Nonlocal Effect

**DOI:** 10.1155/2015/825342

**Published:** 2015-03-01

**Authors:** M. A. De Rosa, M. Lippiello, H. D. Martin

**Affiliations:** ^1^School of Engineering, University of Basilicata, Viale dell'Ateneo Lucano 10, 85100 Potenza, Italy; ^2^Department of Structures for Engineering and Architecture, University of Naples “Federico II”, Via Forno Vecchio 36, 80134 Naples, Italy; ^3^Facultad Regional Reconquista, UTN, Parque Industrial Reconquista, Reconquista, 3560 Santa Fe, Argentina

## Abstract

The Hamilton principle is applied to deduce the free vibration frequencies of a cantilever single-walled carbon nanotube (SWCNT) in the presence of an added mass, which can be distributed along an arbitrary part of the span. The nonlocal elasticity theory by Eringen has been employed, in order to take into account the nanoscale effects. An exact formulation leads to the equations of motion, which can be solved to give the frequencies and the corresponding vibration modes. Moreover, two approximate semianalytical methods are also illustrated, which can provide quick parametric relationships. From a more practical point of view, the problem of detecting the mass of the attached particle has been solved by calculating the relative frequency shift due to the presence of the added mass: from it, the mass value can be easily deduced. The paper ends with some numerical examples, in which the nonlocal effects are thoroughly investigated.

## 1. Introduction

Carbon nanotubes (CNT)—as discovered by Iijima in 1991 (see [1])—have unique electrical, mechanical, and thermal properties, so that they are widely used in a large range of technical areas: nanoelectronics, scanning probes, nanoscale sensors, biomedical devices, and others. From a theoretical point of view, the nanoscale of these structures suggests an atomistic model, but this approach turns out to be very expensive. On the other hand, the usual beam theories (Euler-Bernoulli, Timoshenko, or even higher-order theories [2]) do not capture the influence of the size-effects, because they are inherently scale-free, so that it is usual to adopt the nonlocal elasticity theory, as developed by Eringen in [3, 4]. One of the most important goals of the nanomechanics is to use biosensors in order to detect external deposited masses, and a good mechanical model can be assumed to be a cantilever beam with an attached added mass along the span. It is important to note that the usual hypothesis of a “point mass” is not always justified [5–11], whereas a more realistic model [12] should assume a distributed added mass along a finite portion of the span. For this model, the free vibration frequencies can be calculated according to the classical energy method: the equations of motion and the boundary conditions are derived by applying the Hamilton principle, and the resulting boundary value problem is solved, to give the secular equation, which in turn permits deducing the frequencies.

Sometimes it is necessary to deduce the influence of some control parameter on the free vibration frequencies, so that some parametric curves must be sketched: in these cases the so-called semianalytical (SAN) methods become unvaluable, because they lead to approximate closed-form formulae for the frequencies as functions of the control parameter. In this paper, the added mass will be treated as a control parameter, and the use of two approximate approaches will permit us to examine the variation of the first frequency as a function of the added mass. In the first approach, we generalize a Meirovitch suggestion [13], starting from the equations of motion, in the spirit of Galerkin, whereas in the second method we start from the energies, following a Ritz-like approach. Both methods give close approximations to the true results, so that it is possible to use the parametric curve in order to find the added mass in terms of the frequency shift.

## 2. Analysis of the Problem

**Figure 1:**
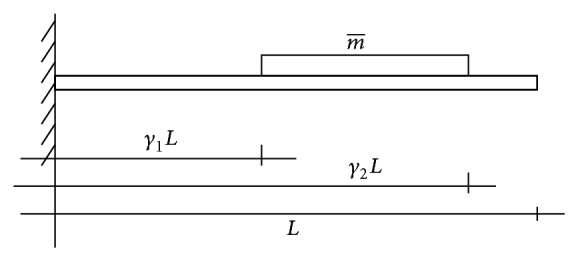
Geometrical properties of the nanotube.

Let us consider the cantilevered nanotube in Figure 1, with span *L*, cross-sectional area *A*, second moment of area *I*, and mass density *ρ*. The well-known Euler-Bernoulli theory for slender beams will be used, so that the Young modulus *E* suffices to define the material properties. Finally, an attached distributed mass $\overset{-}{m}$ is located, between the abscissae *γ*_1_ *L* and *γ*_2_ *L*. In order to take into account the nanoscale effects, the nonlocal elasticity theory has been adopted, as suggested by Eringen.

According to Hamilton principle it is possible to write

(1) $$\begin{array}{c} \overset{t_{2}}{\underset{t_{1}}{\int }}\left(\delta T\left(t\right)-\delta E_{T}\left(t\right)\right)\text{dt}=0, \end{array}$$

where

(2) T=12∫0γ1LρA∂v1(z,t)∂t2dz+12∫γ1Lγ2LρA∂v2(z,t)∂t2dz +12∫γ2LLρA∂v3(z,t)∂t2dz+12∫γ1Lγ2Lm−∂v2(z,t)∂t2dz

is the sum of the kinetic energies of the nanotube in the three sections, *v*_1_ between (0, *γ*_1_ *L*), *v*_2_ between (*γ*_1_ *L*, *γ*_2_ *L*), and *v*_3_ between (*γ*_2_ *L*, *L*), respectively, and of the kinetic energy of the added mass. Moreover,

(3) ET=Le−P=12∫0γ1LEI∂2v1(z,t)∂z22dz +12∫γ1Lγ2LEI∂2v2z,t∂z22dz +12∫γ2LLEI∂2v3(z,t)∂z22dz −∫0γ1LρA∂2v1(z,t)∂t2μ2∂2v1(z,t)∂z2dz −∫γ1Lγ2LρA∂2v2(z,t)∂t2μ2∂2v2(z,t)∂z2dz −∫γ2LLρA∂2v3(z,t)∂t2μ2∂2v3(z,t)∂z2dz

is the total potential energy, *L*_*e*_ is the strain energy of the nanotube, and *P* is the potential energy of the inertial force (*ρA*(∂^2^ *v*(*z*, *t*)/∂*t* ^2^)) due to the additional displacement *μ* ^2^(∂^2^ *v*(*z*, *t*)/∂*z* ^2^) [14]. Here *μ* ^2^ = (*e*_0_ *a*)^2^, where *e*_0_ is a material constant, which has to be defined through experimental results, and *a* is the internal characteristic length of the nanotube.

The first variation of these two energies can be easily calculated so that (1) gives

(4) ∫t1t2∫0γ1LρA∂v1(z,t)∂tδ∂v1(z,t)∂tdz  +∫γ1Lγ2LρA∂v2z,t∂tδ∂v2z,t∂tdz  +∫γ2LLρA∂v3(z,t)∂tδ∂v3(z,t)∂tdz  +∫γ1Lγ2Lm−∂v2(z,t)∂tδ∂v2(z,t)∂tdz  −∫0γ1LEI∂2v1z,t∂z2δ∂2v1z,t∂z2llllllllllllllllllll−μ2ρA∂2v1z,t∂t2δ∂2v1z,t∂z2dz  −∫γ1Lγ2LEI∂2v2z,t∂z2δ∂2v2z,t∂z2llllllllllllllllllll−μ2ρA∂2v2z,t∂t2δ∂2v2z,t∂z2dz  −∫γ2LLEI∂2v3(z,t)∂z2δ∂2v3(z,t)∂z2llllllllllllllllllll−μ2ρA∂2v3z,t∂t2δ∂2v3z,t∂z2dzdt=0.

Integrations by part (see Appendix) lead to a system of three equations of motion:

(5) EI∂4v1z,t∂z4−μ2ρA∂4v1z,t∂z2∂t2+ρA∂2v1z,t∂t2=0,gggggggggggggggggggggggggggggg10<z<γ1L,EI∂4v2z,t∂z4−μ2ρA∂4v2z,t∂z2∂t2+(ρA+M)∂2v2z,t∂t2=0,gggggggggggggggggggggggggggggggγ1L<z<γ2L,EI∂4v3z,t∂z4−μ2ρA∂4v3z,t∂z2∂t2+ρA∂2v3z,t∂t2=0,ggggggggggggggggggggggggggglγ2L<z<L,

together with the following general boundary conditions at the ends:

(6) $$\begin{array}{c} v_{1}\left(0,t\right)=0,\\ \frac{\partial v_{1}\left(0,t\right)}{\partial z}=0,\\ \text{EI}\frac{\partial^{3}v_{3}\left(L,t\right)}{\partial z^{3}}-\mu^{2}\rho A\frac{\partial^{3}v_{3}\left(L,t\right)}{\partial t^{2}\partial z}=0,\\ -\text{EI}\frac{\partial^{2}v_{3}\left(L,t\right)}{\partial z^{2}}+\mu^{2}\rho A\frac{\partial^{2}v_{3}\left(L,t\right)}{\partial t^{2}}=0. \end{array}$$

The boundary conditions for *z* = *γ*_1_ *L* are given by

(7) v1γ1L,t=v2γ1L,t,∂v1γ1L,t∂z=∂v2γ1L,t∂z,μ2ρA∂3v1γ1L,t∂t2∂z−EI∂3v1γ1L,t∂z3−μ2ρA∂3v2γ1L,t∂t2∂z  +EI∂3v2γ1L,t∂z3=0,μ2ρA∂2v1γ1L,t∂t2−EI∂2v1γ1L,t∂z2−μ2ρA∂2v2γ1L,t∂t2  +EI∂2v2γ1L,t∂z2=0,

and finally, at *z* = *γ*_2_ *L*,

(8) v2γ2L,t=v3γ2L,t,∂v2γ2L,t∂z=∂v3γ2L,t∂z,μ2ρA∂3v2γ2L,t∂t2∂z−EI∂3v2γ2L,t∂z3−μ2ρA∂3v3γ2L,t∂t2∂z  +EI∂3v3γ2L,t∂z3=0,μ2ρA∂2v2γ2L,t∂t2−EI∂2v2γ2L,t∂z2−μ2ρA∂2v3γ2L,t∂t2  +EI∂2v3γ2L,t∂z2=0.

The solutions of (5) can be expressed as

(9) $$\begin{array}{c} v_{h}\left(z,t\right)=v_{h}\left(z\right)e^{i\omega t},\;h=1,2,3. \end{array}$$

If the nondimensional abscissa *ζ* = *z*/*L* can be introduced, the system of three equations of motion (5) becomes

(10) ∂4v1ζ∂ζ4+η2Ω4∂2v1ζ∂ζ2−Ω4v1ζ=0 for  0<ζ<γ1,∂4v2ζ∂ζ4+η2Ω4∂2v2ζ∂ζ2−1+λΩ4v2ζ=0ggggggggggggggggggggggggfor  γ1<ζ<γ2,∂4v3ζ∂ζ4+η2Ω4∂2v3ζ∂ζ2−Ω4v3ζ=0 for  γ2<ζ<1,

where the following nondimensional parameters have been introduced:

(11) $$\begin{array}{c} \lambda =\frac{\overset{-}{m}}{\rho A};\;\;\eta =\frac{\mu }{L};\;\;\Omega =\sqrt{\sqrt{\frac{\rho AL^{4}\omega^{2}}{\text{EI}}}}. \end{array}$$

The boundary conditions for clamped-free nanotube are given by

(12) $$\begin{array}{c} v_{1}\left(0\right)=0,\\ \frac{\partial v_{1}\left(0\right)}{\partial \zeta }=0,\\ v_{1}\left(\gamma_{1}\right)=v_{2}\left(\gamma_{1}\right),\\ \frac{\partial v_{1}\left(\gamma_{1}\right)}{\partial \zeta }=\frac{\partial v_{2}\left(\gamma_{1}\right)}{\partial \zeta },\\ \eta^{2}\Omega^{4}\frac{\partial v_{1}\left(\gamma_{1}\right)}{\partial \zeta }+\frac{\partial^{3}v_{1}\left(\gamma_{1}\right)}{\partial \zeta^{3}}-\eta^{2}\Omega^{4}\frac{\partial v_{2}\left(\gamma_{1}\right)}{\partial \zeta }-\frac{\partial^{3}v_{2}\left(\gamma_{1}\right)}{\partial \zeta^{3}}=0,\\ \eta^{2}\Omega^{4}v_{1}\left(\gamma_{1}\right)+\frac{\partial^{2}v_{1}\left(\gamma_{1}\right)}{\partial \zeta^{2}}-\eta^{2}\Omega^{4}v_{2}\left(\gamma_{1}\right)-\frac{\partial^{2}v_{2}\left(\gamma_{1}\right)}{\partial \zeta^{2}}=0,\\ v_{2}\left(\gamma_{2}\right)=v_{3}\left(\gamma_{2}\right),\\ \frac{\partial v_{2}\left(\gamma_{2}\right)}{\partial \zeta }=\frac{\partial v_{3}\left(\gamma_{2}\right)}{\partial \zeta },\\ \eta^{2}\Omega^{4}\frac{\partial v_{2}\left(\gamma_{2}\right)}{\partial \zeta }+\frac{\partial^{3}v_{2}\left(\gamma_{2}\right)}{\partial \zeta^{3}}-\eta^{2}\Omega^{4}\frac{\partial v_{3}\left(\gamma_{2}\right)}{\partial \zeta }-\frac{\partial^{3}v_{3}\left(\gamma_{2}\right)}{\partial \zeta^{3}}=0,\\ \eta^{2}\Omega^{4}v_{2}\left(\gamma_{2}\right)+\frac{\partial^{2}v_{2}\left(\gamma_{2}\right)}{\partial \zeta^{2}}-\eta^{2}\Omega^{4}v_{3}\left(\gamma_{2}\right)-\frac{\partial^{2}v_{3}\left(\gamma_{2}\right)}{\partial \zeta^{2}}=0,\\ \frac{\partial^{3}v_{3}\left(1\right)}{\partial \zeta^{3}}+\eta^{2}\Omega^{4}\frac{\partial v_{3}\left(1\right)}{\partial \zeta }=0,\\ -\frac{\partial^{2}v_{3}\left(1\right)}{\partial \zeta^{2}}-\eta^{2}\Omega^{4}v_{3}\left(1\right)=0. \end{array}$$

The general solutions of (10) are given by

(13) v1ζ=A1cos⁡⁡(αζ)+A2sin⁡(αζ)+A3cos⁡⁡h(αζ) +A4sin⁡hαζ,v2ζ=B1cos⁡⁡α1ζ+B2sin⁡α1ζ+B3cos⁡⁡hβ1ζ +B4sin⁡hβ1ζ,v3ζ=C1cos⁡⁡(αζ)+C2sin⁡(αζ)+C3cos⁡⁡h(βζ) +C4sin⁡h(βζ)

with

(14) α=12η2Ω4+Ω24+η4Ω4;β=12−η2Ω4+Ω24+η4Ω4;α1=12η2Ω4+Ω24+4λ+η4Ω4;β1=12−η2Ω4+Ω24+4λ+η4Ω4.

The twelve constants can be found by imposing the boundary conditions (12). The resulting homogeneous system has nontrivial solutions if and only if the coefficient determinant is zero and the corresponding secular equation has infinite solutions *Ω*_*j*_. The circular frequencies *ω*_*j*_ can be easily deduced, as well as the natural frequencies *f*_*j*_ = *ω*_*j*_/2*π*.

### 2.1. Nonlocal Fundamental Natural Frequency on CNT with Attached Mass-First Method

Starting from the equations of motion (5), it is possible to integrate each of them in their domain, and the resulting integrals can be summed up:

(15) ∫0LEI∂4v(z)∂z4dz+ω2∫0Lμ2ρA∂2v(z)∂z2dz  −ω2∫0LρAv(z)dz−ω2∫γ1Lγ2Lm−v(z)dz=0.

It is now possible to insert a trial function *y*(*z*) [14], leading to

(16) ∫0LEI∂4v(z)∂z4y(z)dz+ω2∫0Lμ2ρA∂2v(z)∂z2y(z)dz  −ω2∫0LρAv(z)y(z)dz−ω2∫γ1Lγ2Lm−v(z)y(z)dz=0.

Two successive integrations by part can be performed:

(17) ∫0LEI∂4vz∂z4yzdz  =EI∂3vz∂z3yz0L−∫0LEI∂3v(z)∂z3∂y(z)∂zdz  =EI∂3vz∂z3yz0L−EI∂2vz∂z2∂yz∂z0L   +∫0LEI∂2vz∂z2∂2yz∂z2dz,ω2∫0Lμ2ρA∂2vz∂z2yzdz  =ω2μ2ρA∂v(z)∂zy(z)0L−ω2∫0Lμ2ρA∂v(z)∂z∂y(z)∂zdz  =ω2μ2ρA∂v(z)∂zy(z)0L−ω2μ2ρAv(z)∂y(z)∂z0L   +ω2∫0Lμ2ρAv(z)∂2y(z)∂z2dz

so that (16) becomes

(18) ∫0L‍EI∂2v(z)∂z2∂2y(z)∂z2dz−ω2∫0L‍ρAv(z)y(z)dz  −ω2∫γ1Lγ2L‍m−v(z)y(z)dz+ω2∫0L‍μ2ρAv(z)∂2y(z)∂z2dz  +EI∂3v(z)∂z3y(z)0L−EI∂2v(z)∂z2∂y(z)∂z0L  +ω2μ2ρA∂vz∂zyz0L−ω2μ2ρAvz∂yz∂z0L=0.

The boundary conditions at the right end permit simplifying the previous equation:

(19) ∫0L‍EI∂2vz∂z2∂2yz∂z2dz+ω2∫0Lμ2ρAv(z)∂2y(z)∂z2dz‍  −ω2∫0L‍ρAv(z)y(z)dz−ω2∫γ1Lγ2L‍m−v(z)y(z)dz  +EI∂3v(L)∂z3y(L)−EI∂2v(L)∂z2∂y(L)∂z  +ω2μ2ρA∂vL∂zy(L)−ω2μ2ρAv(L)∂yL∂z=0,

whereas the free end will be subjected to the following equilibrium conditions:

(20) ω2μ2ρA∂v(L)∂z+EI∂3v(L)∂z3=0,−ω2μ2ρAv(L)−EI∂2vL∂z2=0.

Finally, (19) reduces to

(21) ∫0L‍EI∂2v(z)∂z2∂2y(z)∂z2dz+ω2∫0L‍μ2ρAv(z)∂2y(z)∂z2dz  −ω2∫0L‍ρAv(z)y(z)dz−ω2∫γ1Lγ2L‍m−v(z)y(z)dz=0

and the frequency *ω* ^2^ can be written down, putting *y*(*z*) = *v*(*z*), as

(22) ω2=∫0L‍EI∂2v(z)∂z2∂2v(z)∂z2
dz
  ×∫0L‍μ2ρAv(z)∂2v(z)∂z2dz∫0L‍ρAv2zdz+∫γ1Lγ2L‍m−v2zdz   −∫0L‍μ2ρAv(z)∂2v(z)∂z2dz−1

or, in terms of the nondimensional abscissa *ζ* = *z*/*L*,

(23) ω2=EIL3∫01‍∂2v(ζ)∂ζ2∂2v(ζ)∂ζ2dζ × η2ρAL∫01‍v(ζ)∂2v(ζ)∂ζ2dζρAL∫01‍v2(ζ)dζ+m−L∫γ1γ2‍v2(ζ)dζ   − η2ρAL∫01‍v(ζ)∂2v(ζ)∂ζ2dζ−1.

In order to obtain a satisfactory approximation of the fundamental frequency, we use as approximating function *v*(*ζ*) the exact displacement of the cantilever beam without added mass:

(24) vζ=Cosh1.8751ζ−Cos⁡1.8751ζ −Sinh1.8751−Sin1.8751Cosh1.8751+Cos⁡1.8751 ×Sinh1.8751ζ−Sin1.8751ζ.

The following integrals can be defined [15]:

(25) I1=∫01v2ζdζ=1,I3=∫01‍∂2v(ζ)∂ζ2∂2v(ζ)∂ζ2dζ=12.3623.

For the case of distributed added mass between the abscissae *γ*_1_ *L* and *γ*_2_ *L*, the integral *I*_2_ can be defined as

(26) $$\begin{array}{c} I_{2}=\overset{\gamma_{2}}{\underset{\gamma_{1}}{\int }}v^{2}\left(\zeta \right)\text{d}\zeta \;0\leq \gamma_{1}\leq 1;\;0\leq \gamma_{2}\leq 1, \end{array}$$

and finally, in order to take into account the nonlocal effects, we define the fourth integral:

(27) $$\begin{array}{c} I_{4}=\overset{1}{\underset{0}{\int }}v\left(\zeta \right)\frac{\partial^{2}v\left(\zeta \right)}{\partial \zeta^{2}}\text{d}\zeta =0.858264. \end{array}$$

The fundamental natural frequency can be deduced from (23) in terms of these four integrals as

(28) $$\begin{array}{c} f_{\text{n}1}=\frac{\omega }{2\pi }=\frac{\beta }{2\pi }\sqrt{\frac{I_{3}}{I_{1}+\lambda I_{2}-\eta^{2}I_{4}}}. \end{array}$$

Finally, it is usual to cancel out the first integral, so arriving to the natural frequency,

(29) $$\begin{array}{c} f_{\text{n}1}=\frac{\beta }{2\pi }\frac{C_{k}}{\sqrt{1+\lambda C_{m}-\eta^{2}C_{\text{n}1}}}, \end{array}$$

where

(30) β=EImL4;Ck=I3I1=3.5160;Cn1=I4I1=0.858264;Cm=I2I1

and *C*_*k*_, *C*_n1_, and *C*_*m*_ are the so-called calibration constants.

### 2.2. Nonlocal Fundamental Natural Frequency on CNT with Attached Mass-Second Method

In this approach, let us start from the energy terms:

(31) T=12∫0L‍ρA∂v(z,t)∂t2dz+12∫γ1Lγ2L‍m−∂v(z,t)∂t2dz,ET=Le−P=12∫0L‍EI∂2vz∂z22dz −∫0L‍μ2ρA∂2v(z,t)∂t2∂2v(z,t)∂z2dz

and let us assume the separation of variables

(32) $$\begin{array}{c} v\left(z,t\right)=v\left(z\right)\mathrm{Cos}\left(\omega \text{t}\right) \end{array}$$

so that the energies read

(33) T=ω22∫0LρAvz2dz+∫γ1Lγ2Lm−vz2dzCos⁡2ωt,ET=Le−P=12∫0LEI∂2v(z)∂z22dzSin2(ωt) +ω2∫0Lμ2ρAvz∂2vz∂z2dzSin2ωt.

The maximum kinetic energy will be equal to the maximum total potential energy, so that

(34) 12∫0LEI∂2v(z)∂z22dz+ω2∫0Lμ2ρAv(z)∂2v(z)∂z2dz  =ω22∫0LρAvz2dz+ω22∫γ1Lγ2Lm−vz2dz

and the frequency *ω* ^2^ can be deduced as

(35) ω2=12∫0LEI∂2vz∂z22dz ×−∫0Lμ2ρAvz∂2vz∂z2dz12∫0LρAvz2dz+12∫γ1Lγ2Lm−vz2dz   −∫0Lμ2ρAvz∂2vz∂z2dz−1

or, in terms of the nondimensional abscissa *ζ* = *z*/*L*,

(36) ω2=12EIL3∫01∂2v(ζ)∂ζ2∂2v(ζ)∂ζ2dζ ×η2ρAL∫01v(ζ)∂2v(ζ)∂ζ2dζ12ρAL∫01v2ζdζ+12m−L∫γ1γ2v2ζdζ   −η2ρAL∫01v(ζ)∂2v(ζ)∂ζ2dζ−1.

Let us assume the same approximating function equation (24), so that the following integrals can be calculated:

(37) I1=∫01‍v2ζdζ=1,I3=∫01‍∂2v(ζ)∂ζ2∂2v(ζ)∂ζ2dζ=12.3623;I4=∫01‍v(ζ)∂2v(ζ)∂ζ2dζ=0.858264,

and finally if the added mass is placed between the abscissae *γ*_1_ *L* and *γ*_2_ *L*, the integral *I*_2_ can be obtained as

(38) $$\begin{array}{c} I_{2}=\overset{\gamma_{2}}{\underset{\gamma_{1}}{\int }}‍v^{2}\left(\zeta \right)\text{d}\zeta ;\;0\leq \gamma_{1}\leq 1;\;0\leq \gamma_{2}\leq 1. \end{array}$$

Therefore, an alternative version of the natural frequency can be obtained as

(39) $$\begin{array}{c} f_{\text{n}2}=\frac{\beta }{2\pi }\frac{C_{k}}{\sqrt{1+\lambda C_{m}-\eta^{2}C_{\text{n}2}}} \end{array}$$

with the three calibration constants:

(40) Ck=I3I1=3.51601;Cn2=2I4I1=1.71653;Cm=I2I1.

## 3. Nonlocal Sensor Equations

The sensor equations in the presence of nonlocal elasticity can now be deduced, and the added mass $\overset{-}{m}$ of a biomolecule can be detected by calculating the corresponding CNT frequency shift. In fact, let us start from the natural frequency of the CNT without the added mass:

(41) $$\begin{array}{c} f_{\text{n}0}=\frac{1}{2\pi }C_{k}\beta , \end{array}$$

and let us express the natural frequency, in the presence of the added mass, as (cf. (29) and (39))

(42) $$\begin{array}{c} f_{\text{ni}}=\frac{f_{\text{n}0}}{\sqrt{\left(1+\lambda C_{m}-\eta^{2}C_{\text{ni}}\right)}},\;\;\text{ i }=1,2. \end{array}$$

The frequency shift of the biosensor can be defined as

(43) $$\begin{array}{c} \Delta \text{f}=f_{\text{n}0}-f_{\text{ni}} \end{array}$$

and finally the relative frequency shift is given by

(44) $$\begin{array}{c} \frac{\Delta \text{f}}{f_{\text{n}0}}=\left(1-\frac{1}{\surd \left(1+\lambda C_{m}-\eta^{2}C_{\text{ni}}\right)}\right) \end{array}$$

from which the value of the added mass $\overset{-}{m}$ can be easily obtained:

(45) $$\begin{array}{c} {\left(\frac{\Delta \text{f}}{f_{\text{n}0}}-1\right)}^{2}=\frac{1}{\left(1+\lambda C_{m}-\eta^{2}C_{\text{ni}}\right)}, \end{array}$$

(46) $$\begin{array}{c} \lambda =\frac{1}{C_{m}{\left(\Delta \text{f}/f_{\text{n}0}-1\right)}^{2}}+\frac{C_{\text{ni}}\eta^{2}}{C_{m}}-\frac{1}{C_{m}} \end{array}$$

(47) $$\begin{array}{c} \overset{-}{m}=\frac{\rho A}{C_{m}{\left(\Delta \text{f}/f_{\text{n}0}-1\right)}^{2}}+\frac{C_{\text{ni}}\eta^{2}\rho A}{C_{m}}-\frac{\rho A}{C_{m}} \end{array}$$

and i = 1 for the first approach and i = 2 for the second approach.

## 4. Numerical Examples

### 4.1. First Example

Table 1 shows properties of the cantilever nanotube, which will be used throughout this section. The added distributed mass will be placed from the section *γ*_1_ *L* to the free end, so that *γ*_2_ = 1, and *γ*_1_ will vary from 0.9 to 0.1. In Table 2 the fundamental natural frequency is given for various values of the *γ* = *γ*_2_ − *γ*_1_ = 1 − *γ*_1_ parameter and for increasing values of the nondimensional *η* coefficient (see (11)). The first column gives the fundamental natural frequency in the absence of nonlocal effects. The table has been obtained by solving the system of three differential equations of motion (13), so that the results can be considered “exact.” As can be easily observed, the first fundamental natural frequency increases for increasing values of the *η* parameter, whereas it decreases for increasing values of the *γ* parameter.

The fundamental natural frequency *f*_n1_ is reported in Table 3, as obtained by means of (29) and with *λ* = 1. A numerical comparison with the exact values in Table 1 shows that the relative error is greater for *γ* = 0.1, whereas the results for *γ* = 0.9 almost coincide. At *γ* = 0.1, the relative error varies between 0.035% for *η* = 0.1, 0.34% for *η* = 0.3, and finally 2.36% for *η* = 0.5. Of course, this *η* value can be considered as a limiting case, whereas *η* = 0.1 and *η* = 0.3 are more realistic choices. For example (see [17]), *η* = 0.235 is adopted. In Table 4 the fundamental natural frequency *f*_n2_ is given, as obtained by means of (39) and with *λ* = 1. A numerical comparison with the exact values in Table 2 shows that the relative error is greater than the previous case. More particularly, for *γ* = 0.1 it varies between 0.36% for *η* = 0.1, 2.83% for *η* = 0.3, and finally 8.45% for *η* = 0.5. Therefore, the first method seems to be more reliable than the second one.

### 4.2. Second Example

As a second example, let us suppose that the added mass is distributed along a fixed length, so that *γ* = *γ*_2_ − *γ*_1_ = 0.3, but its real placement along the nanotube is unknown. In Table 5 the first fundamental natural frequency is reported, for different placements of the added mass and for four *η* parameters. The frequencies have been obtained by solving the equations of motion, so that the results can be considered exact, and the nonlocal parameter has been allowed to vary between 0 and 0.3. The fundamental natural frequency increases for increasing values of the nonlocal *η* parameter, and higher values correspond to added masses nearer to the clamped end. The same example is illustrated in Tables 6 and 7, using the approximate formula (29) and the approximate formula (39), respectively. As in the first example, the first method gives better results.

### 4.3. Third Example

**Figure 2:**
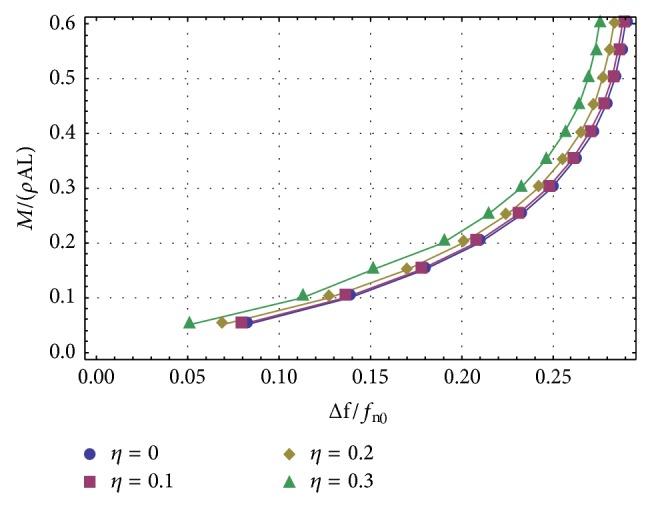
The nondimensional mass ratio *M*/*ρAL*⁡ is plotted against the relative frequency shift—as obtained using (44)—with $M=\overset{-}{m}\left(\gamma_{2}-\gamma_{1}\right)L$. The four curves refer to four different *η* values, *η* = 0 (without nonlocal effects), *η* = 0.1, *η* = 0.2, and *η* = 0.3.

**Figure 3:**
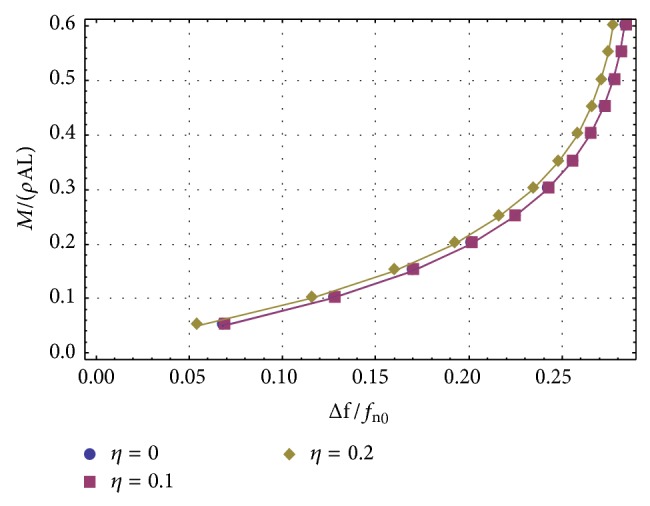
Numerical comparison between two proposed approaches.

Finally, let us address the practical problem of the added mass detection. In order to solve this problem, it is necessary to plot the relationship between the added mass $\overset{-}{m}$ equation (47) and the relative frequency shift equation (44). More precisely, in Figure 2 the nondimensional mass ratio *M*/*ρAL*⁡ is plotted against the relative frequency shift equation (44), with $M=\overset{-}{m}(\gamma_{2}-\gamma_{1})L$, and the four curves refer to four different *η* values, *η* = 0 (without nonlocal effects), *η* = 0.1, *η* = 0.2, and *η* = 0.3. The added mass is placed at the tip of the cantilever nanotube, so that *η*_2_ = 1, whereas (*γ*_2_−*γ*_1_) is allowed to vary between 0.05 and 0.6. The geometrical data of the nanotube are given in Table 1. It is interesting to note that, according to our results, the relative frequency shift decreases for increasing values of the nonlocal coefficient *η*. This should be compared with the different behaviour exhibited by the results given in [17]. The curves in Figure 2 have been drawn using the first approach, because it gives better approximations to the true values. Actually, in Figure 3 we have compared the exact method with the two proposed approaches, for *η* = 0.2, but the curve describing the first approach is undistinguishable from the exact curve.

## 5. Conclusions

The frequency shift between the free vibration frequencies of a cantilever nanotube with, and without, an attached distributed mass has been used, in order to detect the added mass value. It is shown that the size-effects must be taken into account, and the frequencies have to be calculated according to the nonlocal elasticity theory. Three different approaches have been proposed to solve the problem, and their results have been compared for a couple of examples. Moreover, the relative frequency shift decreases, for increasing values of the nonlocal coefficient *η*, so that careful calibration of this coefficient *η* becomes necessary, in order to obtain reliable values of the added mass.

## Figures and Tables

**Table 1:** Nanotube properties (see [16]).

SWCNT properties density	Symbol	Value	Unit
Cross section area	*A*	7.851 10^−19^	m^2^
Radius	*R*	0.5 10^−9^	m
Length	*L*	9 10^−9^	m
Moment of inertia	*I*	4.91 10^−38^	m^4^
Density	*ρ*	2300	Kg/m^3^
Young's modulus	*E*	1000 10^9^	Pa

**Table 2:** The first exact natural frequency (×10^10^) *f*_0_ for various values of the nondimensional length of the added mass and for four increasing values of the nonlocal nondimensional coefficient η.

γ	η = 0	η = 0.1	η = 0.3	η = 0.5
0.1	3.10361	3.11245	3.20756	3.46665
0.2	2.84585	2.85418	2.9260	3.10933
0.3	2.7036	2.71057	2.77009	2.91661
0.4	2.62348	2.6297	2.68251	2.80985
0.5	2.58023	2.58605	2.63527	2.75263
0.6	2.55911	2.56473	2.6122	2.72473
0.7	2.55048	2.55602	2.60277	2.71335
0.8	2.54791	2.55342	2.59996	2.70995
0.9	2.54749	2.55301	2.59951	2.70941

**Table 3:** First approximate fundamental natural frequency (×10^10^) *f*_n1_, as obtained using (29), for various values of the nondimensional length of the added mass and for four increasing values of the nonlocal nondimensional coefficient η.

γ	η = 0	η = 0.1	η = 0.3	η = 0.5
0.1	3.10361	3.11354	3.19659	3.38478
0.2	2.84803	2.85570	2.91937	3.06061
0.3	2.70498	2.71154	2.76586	2.88508
0.4	2.62411	2.63011	2.67959	2.78756
0.5	2.580453	2.586150	2.63315	2.73539
0.6	2.55916	2.56472	2.61054	2.71007
0.7	2.55050	2.5560	2.60135	2.69979
0.8	2.54792	2.55341	2.59861	2.69673
0.9	2.54750	2.55299	2.59817	2.69624

**Table 4:** First exact fundamental natural frequency (×10^10^) *f*_n2_, as obtained using (39), for various values of the nondimensional length of the added mass and for four increasing values of the nonlocal nondimensional coefficient η.

γ	η = 0	η = 0.1	η = 0.3	η = 0.5
0.1	3.10361	3.12357	3.29845	3.75946
0.2	2.84803	2.86343	2.99634	3.32922
0.3	2.70498	2.71816	2.83106	3.10674
0.4	2.62411	2.63614	2.73875	2.98593
0.5	2.58045	2.59188	2.68921	2.92207
0.6	2.55916	2.57032	2.66515	2.89127
0.7	2.55050	2.56154	2.65536	2.87879
0.8	2.54792	2.55893	2.65246	2.87509
0.9	2.54750	2.55851	2.65199	2.87449

**Table 5:** First exact fundamental natural frequency (×10^10^) *f*_0_ for various values of the nondimensional length of the added mass (with γ = 0.3) and for four increasing values of the nonlocal nondimensional coefficient η.

γ_1_	γ_2_	η = 0	η = 0.1	η = 0.2	η = 0.3
0.7	1	2.70498	2.71057	2.73205	2.77009
0.6	0.9	2.90531	2.91317	2.9375	2.98072
0.5	0.8	3.10026	3.10941	3.13780	3.18851
0.4	0.7	3.27557	3.28637	3.31997	3.38053
0.3	0.6	3.41695	3.42952	3.46879	3.54028
0.2	0.5	3.51481	3.52888	3.57304	3.65415
0.1	0.4	3.57016	3.58519	3.63251	3.71994
0.0	0.3	3.59421	3.60970	3.65848	3.74886

**Table 6:** First exact fundamental natural frequency (×10^10^) *f*_1_, as obtained using (29), for various values of the nondimensional length of the added mass (with γ = 0.3) and for four increasing values of the nonlocal nondimensional coefficient η.

γ_1_	γ_2_	η = 0	η = 0.1	η = 0.2	η = 0.3
0.7	1	2.70498	2.71154	2.73154	2.76586
0.6	0.9	2.90556	2.91370	2.93855	2.98142
0.5	0.8	3.10177	3.11168	3.14201	3.19457
0.4	0.7	3.27802	3.28973	3.32563	3.38815
0.3	0.6	3.41894	3.43224	3.47306	3.54445
0.2	0.5	3.51573	3.53019	3.57465	3.65264
0.1	0.4	3.57039	3.585549	3.63215	3.71405
0.0	0.3	3.59424	3.60969	3.65727	3.74092

**Table 7:** First exact fundamental natural frequency (×10^10^) *f*_2_, as obtained using (39), for various values of the nondimensional length of the added mass (with γ = 0.3) and for four increasing values of the nonlocal nondimensional coefficient η.

γ_1_	γ_2_	η = 0	η = 0.1	η = 0.2	η = 0.3
0.7	1	2.70498	2.71816	2.75890	2.83106
0.6	0.9	2.90556	2.92191	2.97270	3.06355
0.5	0.8	3.10177	3.12169	3.18385	3.29624
0.4	0.7	3.27802	3.30157	3.37536	3.51017
0.3	0.6	3.41894	3.44568	3.52982	3.68488
0.2	0.5	3.51573	3.54482	3.63663	3.80690
0.1	0.4	3.57039	3.60087	3.69723	3.87658
0.0	0.3	3.59424	3.62534	3.72373	3.90716

## Appendix

Equation (4), which is reported here for the sake of readability, has to be integrated by part:

(A.1) ∫t1t2∫0γ1L‍ρA∂v1(z,t)∂tδ∂v1(z,t)∂tdz  +∫γ1Lγ2LρA∂v2(z,t)∂tδ∂v2(z,t)∂tdz‍  +∫γ2LL‍ρA∂v3(z,t)∂tδ∂v3(z,t)∂tdz  +∫γ1Lγ2L‍m−∂v2(z,t)∂tδ∂v2(z,t)∂tdz  −∫0γ1L‍EI∂2v1(z,t)∂z2δ∂2v1(z,t)∂z2gggggggggg−μ2ρA∂2v1z,t∂t2δ∂2v1z,t∂z2dz  −∫γ1Lγ2LEI∂2v2z,t∂z2δ∂2v2z,t∂z2gggggggggg−μ2ρA∂2v2z,t∂t2δ∂2v2z,t∂z2dz  −∫γ2LL‍EI∂2v3z,t∂z2δ∂2v3z,t∂z2gggggggggg−μ2ρA∂2v3z,t∂t2gggggggggl×δ∂2v3z,t∂z2dzdt=0.

The first three terms of (A.1) can be treated as follows:

(A.2) ∫0γ1L∫t1t2ρA∂v1(z,t)∂tδ∂v1(z,t)∂tdt dz‍  =∫0γ1LρA∂v1z,t∂tδv1z,tt1t2dz‍   −∫0γ1L∫t1t2ρA∂2v1z,t∂t2δv1z,tdt dz;‍∫γ1Lγ2L∫t1t2ρA∂v2z,t∂tδ∂v2z,t∂tdt dz‍  =∫γ1Lγ2L‍ρA∂v2(z,t)∂tδv2(z,t)t1t2dz   −∫γ1Lγ2L∫t1t2ρA∂2v2(z,t)∂t2δv2(z,t)dt dz;‍∫γ2LL∫t1t2ρA∂v3z,t∂tδ∂v3z,t∂tdt dz‍  =∫γ2LLρA∂v3(z,t)∂tδv3z,tt1t2dz‍   −∫γ2LL∫t1t2ρA∂2v3z,t∂t2δv3z,tdt dz,‍

where we considered the fact that *δ*(*v*_*i*_(*z*, *t*)) = 0 at *t* = *t*_1_ and *t* = *t*_2_.

Quite similarly, the single term of the distributed mass becomes

(A.3) ∫γ1Lγ2L∫t1t2m−∂v2z,t∂tδ∂v2z,t∂tdt dz‍  =∫γ1Lγ2Lm−∂v2z,t∂tδv2z,tt1t2dz‍   −∫γ1Lγ2L∫t1t2m−∂2v2z,t∂t2δv2z,tdt dz.‍

The nonlocal effects are contained into three integrals, which can be integrated as follows:

(A.4) ∫t1t2∫0γ1Lμ2ρA∂2v1z,t∂t2δ∂2v1z,t∂z2dz dt‍  =∫t1t2μ2ρA∂2v1(z,t)∂t2δ∂v1(z,t)∂z0γ1Ldt‍   −∫t1t2∫0γ1Lμ2ρA∂3v1(z,t)∂t2∂zδ∂v1(z,t)∂zdz dt‍  =∫t1t2μ2ρA∂2v1(z,t)∂t2δ∂v1(z,t)∂z0γ1Ldt‍   −∫t1t2μ2ρA∂3v1z,t∂t2∂zδv1z,t0γ1Ldt‍   +∫t1t2∫0γ1Lμ2ρA∂4v1(z,t)∂t2∂z2δv1z,tdz dt;‍∫t1t2∫γ1Lγ2L‍μ2ρA∂2v2(z,t)∂t2δ∂2v2(z,t)∂z2dz dt  =∫t1t2μ2ρA∂2v2(z,t)∂t2δ∂v2(z,t)∂zγ1Lγ2Ldt‍   −∫t1t2∫γ1Lγ2Lμ2ρA∂3v2(z,t)∂t2∂zδ∂v2(z,t)∂zdz dt‍  =∫t1t2μ2ρA∂2v2(z,t)∂t2δ∂v2(z,t)∂zγ1Lγ2Ldt‍   −∫t1t2μ2ρA∂3v2(z,t)∂t2∂zδv2(z,t)γ1Lγ2Ldt‍   +∫t1t2∫γ1Lγ2Lμ2ρA∂4v2(z,t)∂t2∂z2δv2z,tdz dt;‍∫t1t2∫γ2LLμ2ρA∂2v3(z,t)∂t2δ∂2v3(z,t)∂z2dz dt‍  =∫t1t2μ2ρA∂2v3(z,t)∂t2δ∂v3(z,t)∂zγ2LLdt‍   −∫t1t2∫γ2LLμ2ρA∂2v3(z,t)∂t2δ∂v3(z,t)∂zdz dt‍  =∫t1t2μ2ρA∂2v3(z,t)∂t2δ∂v3(z,t)∂zγ2LLdt‍   −∫t1t2μ2ρA∂3v3(z,t)∂t2∂zδv3(z,t)γ2LLdt‍   +∫t1t2∫γ2LLμ2ρA∂4v3(z,t)∂t2∂z2δv3(z,t)dz dt.‍

Finally, the first variation of the strain energy can be treated as usual:

(A.5) −∫t1t2∫0γ1LEI∂2v1z,t∂z2δ∂2v1z,t∂z2dz dt‍  =−∫t1t2EI∂2v1(z,t)∂z2δ∂v1(z,t)∂z0γ1Ldt‍   +∫t1t2∫0γ1LEI∂3v1(z,t)∂z3δ∂v1(z,t)∂zdz dt‍  =−∫t1t2EI∂2v1(z,t)∂z2δ∂v1(z,t)∂z0γ1Ldt‍   +∫t1t2EI∂3v1(z,t)∂z3δv1z,t0γ1Ldt‍   −∫t1t2∫0γ1LEI∂4v1(z,t)∂z4δv1z,tdz dt;‍−∫t1t2‍∫γ1Lγ2L‍EI∂2v2(z,t)∂z2δ∂2v2(z,t)∂z2dz dt  =−∫t1t2‍EI∂2v2(z,t)∂z2δ∂v2(z,t)∂zγ1Lγ2Ldt   +∫t1t2∫γ1Lγ2L‍EI∂3v2(z,t)∂z3δ∂v2(z,t)∂zdz dt  =−∫t1t2EI∂2v2(z,t)∂z2δ∂v2(z,t)∂zγ1Lγ2Ldt‍   +∫t1t2EI∂3v2(z,t)∂z3δv2z,tγ1Lγ2Ldt‍   −∫t1t2∫γ1Lγ2LEI∂4v2(z,t)∂z4δv2z,tdz dt;‍−∫t1t2∫γ2LLEI∂2v3(z,t)∂z2δ∂2v3(z,t)∂z2dz dt‍  =−∫t1t2EI∂2v3(z,t)∂z2δ∂v3(z,t)∂zγ2LLdt‍   +∫t1t2∫γ2LLEI∂3v3(z,t)∂z3δ∂v3(z,t)∂zdz dt‍  =−∫t1t2EI∂2v3(z,t)∂z2δ∂v3(z,t)∂zγ2LLdt‍   +∫t1t2EI∂3v3(z,t)∂z3δv3z,tγ2LLdt‍   −  ∫t1t2∫γ2LLEI∂4v3(z,t)∂z4δv3z,tdz dt‍.

All the previous integrated terms can be collected together, leading to

(A.6) ∫t1t2−∫0γ1LρA∂2v1z,t∂t2δv1z,tdz‍‍   −∫0γ1LEI∂4v1z,t∂z4δv1z,tdz‍   +∫0γ1Lμ2ρA∂4v1(z,t)∂t2∂z2δv1z,tdz‍   −∫γ1Lγ2LρA∂2v2z,t∂t2δv2z,tdz‍   −∫γ1Lγ2LEI∂4v2z,t4δv2z,tdz‍   +∫γ1Lγ2Lμ2ρA‍∂4v2z,t∂t2∂z2δv2z,tdz   −∫γ2LLρA∂2v3z,t∂t2δv3z,tdz‍   −∫γ2LL‍EI∂4v3z,t∂z4δv3z,tdz   +∫γ2LLμ2ρA∂4v3z,t∂t2∂z2δv3z,tdz‍   −∫γ1Lγ2Lm−∂2v2(z,t)∂t2δv2z,tdz‍dt=0;

(A.7) ∫t1t2μ2ρA∂2v1(z,t)∂t2δ∂v1(z,t)∂z0γ1L‍   −μ2ρA∂3v1(z,t)∂t2∂zδv1(z,t)0γ1L   −EI∂2v1(z,t)∂z2δ∂v1(z,t)∂z0γ1L   +EI∂3v1(z,t)∂z3δv1(z,t)0γ1L   +μ2ρA∂2v2(z,t)∂t2δ∂v2(z,t)∂zγ1Lγ2L   −μ2ρA∂3v2(z,t)∂t2∂zδv2(z,t)γ1Lγ2L   −EI∂2v2(z,t)∂z2δ∂v2(z,t)∂zγ1Lγ2L   +EI∂3v2(z,t)∂z3δv2(z,t)γ1Lγ2L   +μ2ρA∂2v3(z,t)∂t2δ∂v3(z,t)∂zγ2LL   −μ2ρA∂3v3(z,t)∂t2∂zδv3(z,t)γ2LL   −EI∂2v3(z,t)∂z2δ∂v3(z,t)∂zγ2LL   +EI∂3v3(z,t)∂z3δv3(z,t)γ2LLdt=0.

Finally, from (A.6) the following equations can be deduced (cf. (4)):

(A.8) EI∂4v1(z,t)∂z4−μ2ρA∂4v1(z,t)∂z2∂t2+ρA∂2v1(z,t)∂t2=0,ggggggggggggggggggggggggggggggg10<z<γ1L,EI∂4v2(z,t)∂z4−μ2ρA∂4v2(z,t)∂z2∂t2+ρA+m−∂2v2(z,t)∂t2=0,ggggggggggggggggggggggggggggggggggggγ1L<z<γ2L,EI∂4v3(z,t)∂z4−μ2ρA∂4v3(z,t)∂z2∂t2+ρA∂2v3(z,t)∂t2=0,ggggggggggggggggggggggggg1ggγ2L<z<L,

whereas the boundary conditions can be deduced from (A.7),

(A.9) v10,t=0,∂v10,t∂z=0,

for *z* = 0 and for *z* = *L*,

(A.10) EI∂3v3(L,t)∂z3−μ2ρA∂3v3L,t∂t2∂z=0,−EI∂2v3L,t∂z2+μ2ρA∂2v3L,t∂t2=0.

The boundary conditions for *z* = *γ*_1_ *L* are

(A.11) v1γ1L,t=v2γ1L,t,∂v1γ1L,t∂z=∂v2γ1L,t∂z,μ2ρA∂3v1γ1L,t∂t2∂z−EI∂3v1γ1L,t∂z3−μ2ρA∂3v2γ1L,t∂t2∂z  +EI∂3v2γ1L,t∂z3=0,μ2ρA∂2v1γ1L,t∂t2−EI∂2v1γ1L,t∂z2−μ2ρA∂2v2γ1L,t∂t2  +EI∂2v2γ1L,t∂z2=0

and those for *z* = *γ*_2_ *L* are

(A.12) v2γ2L,t=v3γ2L,t,∂v2γ2L,t∂z=∂v3γ2L,t∂z,μ2ρA∂3v2γ2L,t∂t2∂z−EI∂3v2γ2L,t∂z3−μ2ρA∂3v3γ2L,t∂t2∂z  +EI∂3v3γ2L,t∂z3=0,μ2ρA∂2v2γ2L,t∂t2−EI∂2v2γ2L,t∂z2−μ2ρA∂2v3γ2L,t∂t2  +EI∂2v3γ2L,t∂z2=0.
